# Influence of the Size and Location of the Perforation on the Anatomical Results of Myringoplasty

**DOI:** 10.7759/cureus.37221

**Published:** 2023-04-06

**Authors:** Ilias Tahiri, Othman El Houari, Amal Hajjij, Mustapha Essaadi, Fouad Benariba

**Affiliations:** 1 Department of Otorhinolaryngology, Mohammed VI University of Health Sciences (UM6SS), Casablanca, MAR; 2 Department of Otolaryngology - Head and Neck Surgery, Faculty of Medicine, Mohammed VI University of Health Sciences (UM6SS), Casablanca, MAR; 3 Department of Otorhinolaryngology, Cheikh Khalifa International University Hospital, Mohammed VI University of Health Sciences (UM6SS), Casablanca, MAR; 4 Department of Otolaryngology - Head and Neck Surgery, Cheikh Khalifa International University Hospital, Faculty of Medicine, Mohammed VI University of Health Sciences (UM6SS), Casablanca, MAR; 5 Department of Otolaryngology - Head and Neck Surgery, Mohammed V Military Training Hospital, Rabat, MAR

**Keywords:** functional results, butterfly technique, perforation size, cartilage graft, myringoplasty

## Abstract

Introduction: Type 1 tympanoplasty (myringoplasty) is the surgical closure of a perforated eardrum. Its purpose is to restore the integrity of the tympanic membrane and to improve hearing in the affected ear. Nowadays, we note the increasing use of cartilage as material for the reconstruction of the tympanic membrane. The main objective of our study is to evaluate the influence of size and perforation site on the results of type 1 tympanoplasties performed in our department.

Materials and methods: We carried out a retrospective study of a series of myringoplasties spread over a period of four years and five months from January 1, 2017, to May 31, 2021. For every patient, we collected data regarding age, sex, perforation size, location, and closure of the tympanic membrane after myringoplasty. The audiological results for air conduction (AC) and bone conduction (BC), as well as air-bone gap reduction following surgery, were noted. Follow-up audiograms were performed at the following intervals: two months, four months, and eight months postoperatively. The frequencies tested included 250, 500, 1000, 2000, and 4000 Hz. Similarly, the air-bone gap was estimated on the mean of all frequencies. A chi-squared test and Mann-Whitney test were used to compare qualitative and quantitative variables, respectively.

Results: A total of 123 myringoplasties were included in this study. Closure of the tympanic membrane was achieved successfully in 85.7% for one-quadrant-size perforations (24 cases), and in 76.2% for two-quadrant-size perforations (16 cases). When 50-75% of the tympanic membrane was absent at the time of diagnosis, full repairment was achieved in 89.6% of the patients (n = 24), and in 85.0% (n = 34) when the perforation was subtotal. Recurrences have not happened more significantly for one location of the tympanic defect compared to another. Indeed, failures for anterior quadrant perforations were 14 whereas other sites represented 19 cases of non-integrated grafts. The audition was significantly improved from pre-operatively (AC mean of 48.7 dBs with ranges from 24 to 90 dBs) to post-operatively (30.7 dBs AC with ranges from 10-80 dBs) (p = 0,002). The average postoperative audiometric Rinne was 18 dBs with a gain of 15.37 dBs.

Discussion: Patients with bilateral perforations (tubal dysfunction, allergic rhinitis) are more likely to develop recurrences. Thus, the series considering many patients operated on twice has high failure rates. Good compliance with anti-allergic treatment and with hygiene rules (in particular ear sealing) is essential for the closure of anterior perforations.

Conclusion: It seems through our study that there is no correlation between the size and location of the perforation and its postoperative closure. Risk factors such as smoking, anemia, intraoperative bleeding, and gastroesophageal reflux are important and determining in the healing process.

## Introduction

Myringoplasty is the surgical closure of a perforated eardrum, whether or not associated with the reconstruction of the ossicular chain [1]. Perforations following chronic otitis media or its sequelae and traumatic perforations are the main indications for myringoplasty. Eardrum perforation has two major consequences for the patient: the opening of the eardrum with a risk of secondary infection and conductive hearing loss due to damage to the integrity of the tympano-ossicular system [2].

Its purpose is to restore the integrity of the tympanic membrane and to improve hearing in the perforated ear. Nowadays, we note the increasing use of cartilage as material for the reconstruction of the tympanic membrane because it has both resistance and better stability and allows excellent auditory results to be obtained [3].

According to the literature, a variation of 71.42% to 92% is reported in the anatomical success rate of myringoplasties [4]. However, certain factors can constitute a source of anatomical failures such as age, tubal dysfunction, inflammation of the middle ear, type of graft, and surgical technique [4]. The main objective of our study is to evaluate the influence of size and perforation site on the results of type 1 tympanoplasties performed in our department.

## Materials and methods

We carried out a retrospective study of a series of myringoplasties spread over a period of four years and five months from January 1, 2017, to May 31, 2021. For every patient, we collected data regarding age, sex, perforation size, location, and closure of the tympanic membrane after myringoplasty. The audiological results for air conduction (AC) and bone conduction (BC), as well as air-bone gap reduction following surgery, were noted.

We included all patients operated on for a simple tympanic perforation. We excluded patients operated on for chronic cholesteatomatous otitis, patients with incomplete or unrecovered files, patients lost to follow-up, and operated patients who did not benefit from a postoperative audiometric evaluation.

Surgeries were performed under general anesthesia by five different surgeons in our department. Both retroauricular external and endoscopic (transcanal) approaches were used to operate on patients with the underlay technique. Cartilage was collected in the conchal area.

We operated on all of our patients under general anesthesia. Before the incision, we proceeded to the infiltration of the retroauricular skin and that of the conduit with a 2% xylocaine adrenaline solution. This procedure aims to reduce bleeding and allows for better dissection of the planes. The graft material used was conchal cartilage devoid of the perichondrium. It was removed after successive incisions of the skin and retroauricular muscle, then release of the cartilage with scissors. A tympano-meatal flap was then lifted to detach the posterior part of the annulus. The opening of the tympanic cavity was followed by the detachment of the malleus from its tympanic attachment. The graft was then slid using the supramartellar subfibrous technique. This procedure then guides for epidermization and closure of the perforation.

Follow-up audiograms were performed at the following intervals: two months, four months, and eight months postoperatively. The frequencies tested included 250, 500, 1000, 2000, and 4000 Hz. A tonal audiometric loss (TAL) average was calculated by the audiometry software. Similarly, the air-bone gap was estimated on the mean of all frequencies.

The size of the perforation was assessed with reference to that of the tympanic quadrants. The eardrum is divided into four quadrants of almost equal dimensions by two lines: the first follows the axis of the handle of the malleus and the second is perpendicular to the first one, passing by the umbo. Thus, the tympanic membrane is divided into four sections or quadrants of almost equal dimensions: anteroinferior, anterosuperior, posterosuperior, and posteroinferior.

Percentage values ​​were used to describe qualitative characteristics, while mean and standard deviation were used for quantitative data. A chi-squared test and Mann-Whitney test were used to compare qualitative and quantitative variables, respectively. Data collection was carried out on archived patients’ files according to the inclusion and exclusion criteria. We recorded the results on a datasheet in Microsoft Office Excel 2019 (Microsoft Corporation, Redmond, WA). This study was carried out while respecting the secrecy and anonymity of patients, as well as the confidentiality of their information. Statistical analysis was performed using SPSS software (IBM Corp., Armonk, NY).

## Results

From January 1, 2017, to May 31, 2021, 123 patients were involved in this retrospective study. The average age of our patients was 40 ± 15.3 years old, with extremes ranging from four to 81 years old. A female predominance with 77 women (63%) and 46 men (37%) was found for sex distribution (p = 0.04). The retroauricular incision was the most commonly used approach in 113 patients (91.87%), followed by the transmeatal incision under endoscopic guidance in 10 patients (8.13%).

The consultation period ranged from six months to several years and the symptoms that motivated the consultation were otorrhea in 114 cases (92.6%), hearing loss in 110 cases (89.4%), and tinnitus in 56 cases (45.5%). The perforation was one-quadrant-size (<25% of the tympanic membrane) in 28 cases (22.8%) and two-quadrant-size in 21 cases (17.1%). In 29 cases (23.6%), it was subtotal (three quadrants size) and in 40 cases (32.5%), it was a total defect. The graft integration rate was in 90 patients (73.2%). Failed surgeries (i.e., 26.8%) occurred mostly because of the inflammatory component of the middle ear cavity preoperatively (n = 15).

Closure of the tympanic membrane was achieved successfully in 85.7% for one-quadrant-size perforations (24 cases), and in 76.2% for two-quadrant-size perforations (16 cases). When 50-75% of the tympanic membrane was absent at the time of diagnosis, full repairment was achieved in 89.6% of the patients (n = 24), and in 85.0% (n = 34) when the perforation was subtotal (Table 1).

**Table 1:** Tympanic closure according to the size of the perforation

Perforation size	Number of ears	Closure rate after myringoplasty	Failures
1 quadrant (<25%)	28	24 (85.7%)	4
2 quadrants (25-50%)	21	16 (76.2%)	5
3 quadrants (50-75%)	29	26 (89.6%)	3
4 quadrants (75-100%)	40	34 (85.0%)	6

Recurrences have not occurred more significantly for one location of the tympanic defect compared to another. Indeed, failures for anterior quadrant perforations were 14 whereas other sites represented 19 cases of non-integrated grafts (p = 0.318).

The postoperative audiogram was carried out systematically for all the patients at eight months postoperative to evaluate the functional results of the surgery. The evaluation of hearing in the patients at follow-up was marked by an improvement in hearing in 76 patients (84.4%), the same pre and postoperative hearing in 11 patients (12.2%), and worsening in three patients (3.4%).

**Figure 1:**
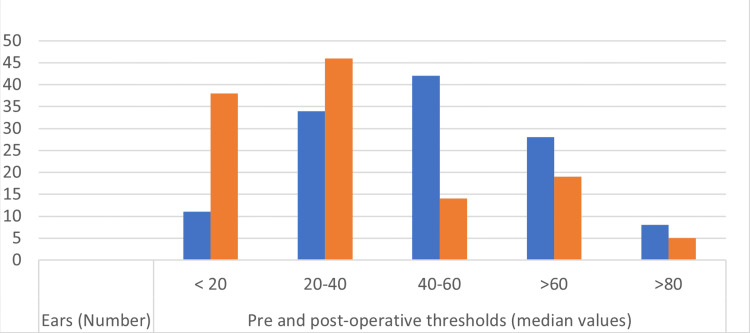
Hearing threshold intervals according to the number of patients Preoperative thresholds are in blue. Postoperative thresholds are in orange.

For 38 patients (30.9%), the final AC threshold was <20 dBs (mean value), between 20 and 40 dBs for 34 (27.7%), over 60 dBs for 19 (15.4%), and over 80 dBs for five patients (40.7%) (Figure 1).

Concerning BC, it was measured at 15.3 dBs (0-60 dBs) before surgery and 12.7 dBs (0-60 dBs) after surgery (p = 0.488). The audition was significantly improved from preoperatively (AC mean of 48.7 dBs with ranges from 24 to 90 dBs) to postoperatively (30.7 dBs AC with ranges from 10 to 80 dBs) (p = 0.002). The average postoperative audiometric Rinne was 18 dBs with a gain of 15.37 dBs. Before myringoplasty, its mean was 33.4 dBs (5-65 dBs) (Table 2). We did observe that the location of the perforation was not positively correlated to the importance of audition gain (p = 0.514).

**Table 2:** Average postoperative hearing gains Minimum and maximum values are ​​in brackets.

Hearing findings	Before surgery	Postoperative
Air conduction	48.7 dBs (24-90)	30.7 dBs (10-80)
Bone conduction	15.3 dBs (0-60)	12.7 dBs (0-60)
Air-bone gap	33.4 dBs (5-65)	18.0 dBs (3-50)

## Discussion

The cartilage graft myringoplasty technique is previously described in the literature. It confers satisfactory results with the restoration of tympanic permeability ranging from 71.4% to 92%, according to the authors [5]. In this study, we opted for a one-piece cartilage graft, whereas other authors perform a "butterfly inlay" technique. They sample the graft in multiple pieces with posterior bone support [6].

We opted for the classical technique since the second favors the occurrence of cholesteatoma. Indeed, the defect present between the pieces of cartilage could see invagination of the epidermal pearls of the cutaneous layer of the eardrum [7]. Other authors advocate a fascia graft devoid of perichondrium to reduce the risk of infection. However, it appears through comparative studies that the success rate of the graft is significantly lower with this technique [8]. On the other hand, the auditory functional results seem similar to the two techniques [9].

In our study, tympanic closure was observed with a high success rate for defects involving more than 50% of the surface of the eardrum (89.6% for large perforations and 85.0% for subtotal perforations, respectively). When less than 50% of the pars tensa was affected, an average surgical success of 79.8% was described. Thus, it appeared that there was no relationship between the size of the perforation and the success of graft adhesion. These data are supported by studies conducted by several authors [10-12].

In contrast, other studies have shown a positive correlation between the size of the perforation and the percentage of success with 74% closure in small perforations and 56% in large ones [13], 90% closure in small perforations and 54.5% in large ones [14], and 91% closure in small perforations and 75% in large ones [15]. It appears that there is no obvious reason for the discrepancy between these results. However, patients with bilateral perforations (tubal dysfunction, allergic rhinitis) are more likely to develop recurrences. Thus, the series considering many patients operated on twice have high failure rates [16].

Also, risk factors such as smoking, anemia, and gastroesophageal reflux are important and determining in the healing process [17].

On the other hand, the correlation between the site (anterior) and less successful closure of the defect has been reported in many studies [18]. Indeed, the proximity of the auditory tube as well as the strong attachment of the annulus to the level of the sulcus prevents physiological epidermization. At the level of the posterosuperior quadrant, the thin character of the annular insertion and the rich vascularization of this zone allow better healing [19].

In our study, there was no significant difference in closure between anterior and posterior tympanic perforations (for 18 cases, eight are posterior and 10 are anterior). This could be explained by the very good compliance with anti-allergic treatment (tubal dysfunction) in some patients and poor compliance with hygiene rules (in particular ear sealing) in others. Indeed, for subjects with chronic rhinosinusitis, anterior tympanic defects and their recurrences are much more frequent than posterior ones.

A new procedure for performing myringoplasty is the butterfly technique. This surgery does not require the making of a tympano-meatal flap but only the freshening of the perforation edges under endoscopy [20]. The tragal cartilage graft is trimmed to fit into the perforation. Part of this cartilage is lateral to the eardrum while the other is medial and rests in the tympanic cavity.

In addition, this technique would make it possible to achieve better hearing gains by reinforcing the columellar effect. We agree with this observation, but we still trust in the one-piece cartilage technique given the very good functional improvement of our patients (18 dBs post-surgery). These postoperative findings are similar to those of the "butterfly onlay" technique. Nevertheless, it should be noted that the thickness of the cartilage must necessarily be less than or equal to 0.4 mm for the classic technique so as not to hinder air conduction [20].

The limitation of our study was that punctiform perforations were not included, given their very high success in surgical closure. Also, a comparative study between the results of our technique, the "butterfly onlay" surgery, and other types of grafts (fascia, perichondrium, etc.) would be useful in the future.

## Conclusions

The one-piece cartilage graft myringoplasty is a reliable surgical technique for tympanic closure. The observed non-correlation between the size of the perforation and its surgical closure is explained by other risk factors (inflammation of the eardrum, infection, anemia, etc.). It appears, through our study, that compliance with anti-allergic treatment, especially for anterior perforations, is an important factor in the non-recurrence of the perforation.
